# p53 Interaction with JMJD3 Results in Its Nuclear Distribution during Mouse Neural Stem Cell Differentiation

**DOI:** 10.1371/journal.pone.0018421

**Published:** 2011-03-31

**Authors:** Susana Solá, Joana M. Xavier, Daniela M. Santos, Márcia M. Aranha, Ana L. Morgado, Kristen Jepsen, Cecília M. P. Rodrigues

**Affiliations:** 1 Research Institute for Medicines and Pharmaceutical Sciences (iMed.UL), Faculty of Pharmacy, University of Lisbon, Lisbon, Portugal; 2 Howard Hughes Medical Institute, Department of Medicine, University of California San Diego, San Diego, California, United States of America; Hertie Institute for Clinical Brain Research and German Center for Neurodegenerative Diseases, Germany

## Abstract

Conserved elements of apoptosis are also integral components of cellular differentiation. In this regard, p53 is involved in neurogenesis, being required for neurite outgrowth in primary neurons and for axonal regeneration in mice. Interestingly, demethylases regulate p53 activity and its interaction with co-activators by acting on non-histone proteins. In addition, the histone H3 lysine 27-specific demethylase JMJD3 induces ARF expression, thereby stabilizing p53 in mouse embryonic fibroblasts. We hypothesized that p53 interacts with key regulators of neurogenesis to redirect stem cells to differentiation, as an alternative to cell death. Specifically, we investigated the potential cross-talk between p53 and JMJD3 during mouse neural stem cell (NSC) differentiation. Our results demonstrated that JMJD3 mRNA and protein levels were increased early in mouse NSC differentiation, when JMJD3 activity was readily detected. Importantly, modulation of JMJD3 in NSCs resulted in changes of total p53 protein, coincident with increased ARF mRNA and protein expression. ChIP analysis revealed that JMJD3 was present at the promoter and exon 1 regions of ARF during neural differentiation, although without changes in H3K27me3. Immunoprecipitation assays demonstrated a direct interaction between p53 and JMJD3, independent of the C-terminal region of JMJD3, and modulation of p53 methylation by JMJD3-demethylase activity. Finally, transfection of mutant JMJD3 showed that the demethylase activity of JMJD3 was crucial in regulating p53 cellular distribution and function. In conclusion, JMJD3 induces p53 stabilization in mouse NSCs through ARF-dependent mechanisms, directly interacts with p53 and, importantly, causes nuclear accumulation of p53. This suggests that JMJD3 and p53 act in a common pathway during neurogenesis.

## Introduction

It has been shown that components of the apoptosis process are pivotal for differentiation [1]. We have recently reported the involvement of specific apoptosis-related proteins in mouse neural stem cell (NSC) differentiation [2]. p53 phosphorylation and transcriptional activation increase throughout differentiation of mouse NSCs, with no evidence of apoptosis. Notably, p53 knockdown delays mouse NSC differentiation. Others have shown that inactivation of p53 and phosphatase and tensin homolog deleted on chromosome ten (PTEN) promotes the undifferentiated state of neural precursor cells [3], and that p53 is required for neurite outgrowth in primary neurons and for axonal regeneration in mice [4]. More recently, several studies have investigated how disruption of the p53 network enhances cellular pluripotency [5], [6], [7], [8], [9], although without a clear consensus on mechanisms by which p53 induces differentiation [10]. Indeed, the specific targets and cofactors of p53 during neurogenesis are still largely unknown. Once identified, they might be strategically manipulated to increase neural fate, as an alternative to cell death, and improve the efficiency of stem cell production. It is possible that p53 may sense epigenetic changes that accompany reprogramming of cells to either differentiated or undifferentiated stages. In this respect, p53 could be upregulated by events that reverse the Polycomb group (PcG) silencing mechanism and/or interfere with members of the PcG machinery. Finally, post-translation modifications (PTMs), including ubiquitination and methylation may allow p53 to lead the specific outcome of neural differentiation [11]. DNA methylation is a dynamic epigenetic mark that undergoes extensive changes during cellular differentiation. Analysis of embryonic stem cells revealed aberrant hypermethylation during extended proliferation *in vitro*, in a pattern reminiscent of that reported in some tumors [12].

JMJD3, identified as H3K27me3 demethylase, controls the expression of key regulators and markers of neurogenesis, and is required for commitment to the neural lineage [13], [14]. Nevertheless, the precise molecular targets of JMJD3 remain largely uncharacterized. The regulation of JMJD3 appears to be highly gene- and context- specific, suggesting interplay with specific molecules to promote fine-tuning more than the on/off alternation of methylation status. It is possible that cellular events responsible for JMJD3 activity are similar to those observed during cell cycle arrest at G1/S or G2/M, or senescence.

The INK4a/ARF tumor suppressor locus, a key executioner of cellular senescence is silenced by several methylases of the PcG of transcription repressors. JMJD3 acts as a tumor suppressor molecule by derepressing this specific locus, thus increasing the expression of INK4a and ARF in human and mouse fibroblasts, respectively [15], [16]. These observations raise the possibility that JMJD3 may contribute to increased p53 levels and activity during neuronal differentiation through an ARF-dependent manner, since ARF inhibits p53 ubiquitination and subsequent degradation [17]. In fact, the ARF-p53 pathway attenuates self-renewal and promotes differentiation of NSCs [18], and the PcG gene *Bmi1* promotes cell proliferation and neural stem cell self-renewal by repressing the INK4a/ARF locus [19]. In addition, it has been suggested that p53 communicates with sensors of cell epigenetic changes and responds by preventing dedifferentiation. Indeed, p53 influences histone H3 acetylation [20]. Finally, several studies have established protein methylation as a novel mechanism of p53 regulation [21]. Several histone lysine methyltransferases methylate p53 at specific C-terminal lysines, preventing or inducing p53 interaction with its co-activators. p53 directly interacts with lysine-specific demethylase 1 (LSD1) to alter chromatin structure and confer developmental repression of a specific tumor marker [22]. Thus, it is not surprising that in response to specific cellular differentiation signals, JMJD3 may directly interact with p53 to fine-tune its activity, thus influencing the equilibrium between neuronal differentiation and cell death. In this regard, it is crucial to understand the apoptosis mechanisms that may overlap with neuronal differentiation pathways. Here, we elucidate the cross-talk between p53 and the demethylase JMJD3 during mouse NSC differentiation. Our data strongly suggest that p53 cellular distribution and function is modulated by direct JMJD3-dependent demethylation of p53 during neurogenesis. This corroborates observations that p53 is dynamically regulated by lysine methylation and demethylation processes, which in turn confer distinct regulatory characteristics to p53.

## Materials and Methods

### Ethics

Mouse neural stem cells used in this study were obtained from Dr. Reynold's Laboratory at the University of Queensland, Brisbane, Australia, and provided by Dr. Low's Laboratory at the University of Minnesota, Minneapolis, MN, USA. The Animal Ethical Committee at the Faculty of Pharmacy, University of Lisbon, Portugal waived the need for approval.

### Mouse NSC Culture and Differentiation

Mouse NSCs containing a constitutively expressed marker for green fluorescence protein (GFP) were used to investigate the process of neuronal differentiation. Cells were obtained from E14 mouse embryo central nervous system and cultured as previously described [23], [24]. Mouse NSCs were maintained as neurospheres in undifferentiating conditions, a serum-free, 1∶1 mix of DMEM/F12 (Invitrogen Corp., Grand Island, NY) with 1X N-2 supplement (Invitrogen Corp.), 20 ng/ml EGF, 20 ng/ml β-FGF (R & D Systems Inc., Minneapolis, MN), and 1% penicillin-streptomycin (Invitrogen Corp.), at 37°C in humidified atmosphere of 5% CO_2_. Subculture was at day 7 with mechanical dissociation of neurospheres. Plating was at 1×10^5^ cell/ml density on T75 flasks, and half of the culture medium was changed after 3 days. The differentiation of mouse NSCs *in vitro* was induced by culturing dissociated cells in differentiation medium containing DMEM/F12 with 1X N-2 supplement, 100 ng/ml β-FGF, 10% FBS (Invitrogen Corp.), 500 nM all-*trans* retinoic acid (Sigma Chemical Co., St. Louis, MO), 50 µM taurine (Sigma Chemical Co.), 10 ng/ml TGF-β2 (R & D Systems Inc.) and 1% penicillin-streptomycin, in tissue culture plates pre-coated with poly-D-lysine (Sigma Chemical Co.). The culture medium was changed every 3 days. Differentiated cells at 1×10^5^ cells/ml were fixed at 0, 6, 12, 24 and 48 h and processed for immunostaining and evaluation of apoptosis. For western blot analysis, cultures at 5×10^5^ cells/ml were processed to assess the role of JMJD3 in modulating p53 during neuron differentiation. All experiments were performed using adherent cells only to exclude detached apoptotic cells.

### Transfections

Mouse NSCs were transfected with Flag-JMJD3 or Flag-JMJD3mut overexpression plasmids to amplify JMJD3 expression. The Flag-JMJD3 construct was cloned by inserting full-length mouse JMJD3 cDNA in frame into p3xFLAG CMV-10 (Sigma Chemical Co.) vector within *Hind*III and *Bam*HI sites. The Flag-JMJD3mut was generated by removing the carboxy-terminus 410 amino acids, which include the jumonji C domain. Three h after plating, the culture differentiation medium was changed to medium without 1% penicillin-streptomycin. Mouse NSCs were transfected using Lipofectamine™ Transfection Reagent (Invitrogen Corp.), according to the manufacturer's instructions. For controls, cells were incubated with transfection agents at the same concentrations and times, in absence of any plasmid (mock). To assess transfection efficiency, Flag protein levels were determined by Western blot. The effect of JMJD3 overexpression throughout the differentiation process was investigated in attached cells either harvested for immunolotting and real time RT-PCR, or used for immunocytochemistry, deoxynucleotidyltransferase-mediated dUTP nick end labeling (TUNEL) and Hoechst assays after 0, 6, 12, 24 and 48 h of differentiation.

### Immunocytochemistry

Mouse NSCs were fixed with 4% paraformaldehyde in phosphate-buffered saline (PBS) for 30 min during differentiation. Cells were then blocked for 1 h at room temperature in PBS containing 0.1% Triton-X-100, 1% FBS, and 10% normal donkey serum (Jackson Immuno Research Laboratories, Inc., West Grove, PA). Subsequently, cells were incubated with either polyclonal antibodies to JMJD3 (1∶50) (KDM6B, Abcam plc, Cambridge, UK), or monoclonal antibodies to p53 (1∶50) (Pab 240, Santa Cruz Biotechnology, Santa Cruz, CA) in blocking solution, overnight at 4°C. After three washes with PBS, cells were incubated with the Alexa Fluor 594-anti-rabbit (1∶200) or the Alexa Fluor 568-anti-mouse (1∶200) conjugated secondary antibodies (Invitrogen Corp.) for 2 h at room temperature. Negative controls, without primary antibodies were also performed. Additionally, cells were incubated with Hoechst dye for nuclear staining. The cellular distribution of JMJD3 and p53 was visualized using an Axioskop fluorescence microscope (Carl Zeiss, Jena, Germany). Total GFP-positive cells were counted on a computer screen grid from at least four random fields (x400).

### Evaluation of Apoptosis

Hoechst labeling and TUNEL staining of mouse NSCs were used to detect apoptotic nuclei. In brief, for morphologic evaluation of apoptosis, the medium was gently removed at the indicated times with minimal detachment of cells. Attached cells were fixed with 4% paraformaldehyde in PBS, pH 7.4, for 10 min at room temperature, incubated with Hoechst dye 33258 (Sigma Chemical Co.) at 5 µg/ml in PBS for 5 min, washed with PBS and mounted using PBS:glycerol (3∶1, v/v). Fluorescent nuclei were scored blindly and categorized according to the condensation and staining characteristics of chromatin. Normal nuclei showed non-condensed chromatin dispersed over the entire nucleus. Apoptotic nuclei were identified by condensed chromatin, contiguous to the nuclear membrane, as well as nuclear fragmentation of condensed chromatin. Three random microscopic fields per sample were counted and mean values expressed as the percentage of apoptotic nuclei. Apoptotic cells were also quantified using the TUNEL assay. Cells were fixed with 4% formaldehyde and processed using an ApopTag *in situ* apoptosis detection kit (Chemicon Int., Temecula, CA), according to the manufacturer's instructions. The number of TUNEL-positive cells was counted on a computer screen grid from at least three random fields (x400). Positive controls were included, which corresponded to mouse NSCs treated with staurosporine and normal female rodent mammary gland tissue with extensive apoptosis. The negative control was performed without active terminal transferase, but including permeabilization, to control for nonspecific incorporation of nucleotides or for nonspecific binding of enzyme-conjugate.

### Total, Cytosolic, and Nuclear Protein Extraction

For total protein extracts, adherent mouse NSCs were lysed in ice-cold buffer (10 mM Tris-HCl, pH 7.6, 5 mM MgCl_2_, 1.5 mM KAc, 1% Nonidet P-40, 2 mM DTT, and protease inhibitor cocktail tablets Complete (Roche Applied Science, Mannheim, Germany)) for 30 min, and then homogenized with 20 strokes in a loose fitting Dounce. The lysate was centrifuged at 3200 *g* for 10 min at 4°C, and the supernatant recovered. For nuclear and cytosolic extracts, cells were lysed with hypotonic buffer (10 mM Tris-HCl, pH 7.6, 5 mM MgCl_2_, 1.5 mM KAc, 2 mM DTT, and protease inhibitors), homogenized with 20 strokes in a loose fitting Dounce, and centrifuged at 500 *g* for 10 min at 4°C. The cytosolic proteins were recovered in the supernatant, while the nuclear pellet was washed in buffer containing 10 mM Tris-HCl, pH 7.6, 5 mM MgCl_2,_ 0.25 M sucrose, 0.5% Triton X-100, and protease inhibitors, then resuspended and sonicated in buffer containing 10 mM Tris-HCl, pH 7.6, 0.25 M sucrose with protease inhibitors. Finally, the suspension was centrifuged through 0.88 M sucrose at 2000 *g* for 20 min at 4°C, and nuclear proteins were recovered in the supernatant.

### Histone Purification

Histone purification was performed using the acid extraction protocol. Briefly, cells were collected and washed in PBS and lysed with hypotonic lysis buffer (10 mM Tris-HCl, pH 8, 1 mM KCl, 1.5 mM MgCl_2_, 1 mM DTT and protease inhibitors) for 30 min at 4°C. Intact nuclei were recovered by centrifuging at 10000 *g* for 10 min at 4°C, and the supernatant discarded. Nuclei were resuspended in 0.4 N H_2_SO_4_, and incubated on rotator at 4°C overnight. Nuclear debris were removed by centrifuging at 16000 *g* for 10 min at 4°C, and the supernatant containing histones was transferred into a fresh tube. 100% TCA was added drop by drop to histone solution, mixed and incubated on ice for 30 min. Histones were recovered after centrifugation at 16000 *g* for 10 min at 4°C. The histone pellet was washed twice with ice-cold acetone and air-dried for 20 min at room temperature. Histones were finally dissolved in water and transferred into a new tube.

### Immunoblotting

Protein levels of JMJD3-Flag, H3K27me3, p53, β-III tubulin, ARF and JMJD3 were determined by Western blot, using either primary polyclonal antibody reactive to H3K27me3 (Abcam plc, Cambridge, UK) and JMJD3 (RB10082, Abgent Inc, San Diego, CA) or primary mouse monoclonal antibodies to Flag (M2, Sigma Chemical Co.), p53 (Pab 240, Santa Cruz Biotechnology), ARF (CDKN2A, Abcam plc) and β-III tubulin (Tuj1, Covance, Princeton, New Jersey) as well as secondary antibodies conjugated with horseradish peroxidase (Bio-Rad Laboratories, Hercules, CA, USA). The specificity of p53 antibody was assessed by Western blot in p53 silenced cells (Fig. S1). Membranes were processed for protein detection using Super Signal™ substrate (Pierce, Rockford, IL). GAPDH was used to control for lane loading, while total histone H3 was used as a marker for nuclear protein extraction. Protein concentrations were determined using the Bio-Rad protein assay kit according to the manufactur's specifications.

### Immnunoprecipitation

The physical association of JMJD3 and p53 was detected by immunoprecipitation analysis. In brief, wholecell extracts were prepared by lysing cells by means of sonication in lysis buffer (50 mM Tris-HCl pH 7.4, 180 mM NaCl, 1 mM EDTA, 0,5% Triton X-100, and protease inhibitors). Immunoprecipitation experiments were carried out using the monoclonal antibody to p53 (Pab 240, Santa Cruz Biotechnology) and the Ezview Red Protein G Affinity Gel (Sigma Chemical Co.). Typically, 500 g of lysate was incubated with 1 µg of primary mouse monoclonal antibody to p53 overnight at 4°C. Immunoblots were then probed with the rabbit polyclonal Flag (M2, Sigma Chemical Co.) or JMJD3 (KDM6B, Abcam plc) antibodies. p53 expression was determined in the same membrane after stripping off the immune complex for the detection of Flag-JMJD3 or JMJD3. Finally, immunoprecipitation assays using mouse monoclonal antibodies reactive to IgG showed no detectable association with either JMJD3 or p53. Methylated levels of p53 after JMJD3 or JMJD3mut transfections were detected by immunoprecipitation analysis in denaturing conditions. In brief, whole cell extracts were prepared, and immunoprecipitation experiments were carried out using the monoclonal antibody to p53 and the Ezview Red Protein G Affinity Gel as above Immunoblots were then probed with the rabbit polyclonal methylated lysine (MeK) (Abcam plc) antibody. p53 expression was determined in the same membrane after stripping off the immune complex for the detection of MeK. Finally, the results of MeK after p53 immunoprecipitation were normalized with those obtained using mouse monoclonal antibodies reactive to IgG immunoprecipitation assays as well as with p53 total levels.

### RNA Isolation and RT-PCR

Total RNA was extracted from mouse NSCs using the TRIZOL reagent (Invitrogen Corp.). Transcript expression of JMJD3 and Pax6 were determined by RT-PCR. For RT-PCR, 5 µg of total RNA was reverse-transcribed using oligo(dT) (Integrated DNA Technologies Inc., Coralville, IA) and SuperScript II reverse transcriptase (Invitrogen Corp.). Specific oligonucleotide primer pairs were incubated with cDNA template for PCR amplification using the Expand High Fidelity^PLUS^ PCR System (Roche Applied Science). The following sequences were used as primers: JMJD3 sense 5′-CCCCCATTTCAGCTGACTAA-3′; JMJD3 antisense 5′-CTGGACCAAGGGGTGTGTT-3′; Pax6 sense 5′-AACACCAACTCCATCAGTTC-3′; Pax6 antisense 5′-ATCTGGATAATGGGTCCTCT-3′; GAPDH sense 5′- ATTCAACGGCACAGTCAAGG-3′; and GAPDH antisense 5′- TGGATGCAGGGATGATGTTC-3′. The product of the GAPDH RNA was used for endogenous normalization after testing several housekeeping genes for appropriate control normalization.

### Chromatin Immunoprecipitation (ChIP)

Mouse NSCs were fixed at 0, 6, 24 and 48 h after differentiation with 0.8% formaldehyde for 10 min at room temperature. After cross-linking, the reaction was quenched with 0.125 M of glycine for 10 min at room temperature. Cells were washed twice with ice-cold PBS, pelleted by centrifugation, resuspended in 1 mL of cell lysis buffer (5 mM PIPES pH 8.0, 85 mM KCl, 0.5% Igepal and 1X protease inhibitor cocktail), and incubated 30 min at 4°C. After centrifugation the nuclei were resuspended in nuclei lysis buffer (50 mM Tris-HCl pH 8.1, 10 mM EDTA, 1% SDS and 1X protease inhibitor cocktail) and incubated for 10 min on ice. The soluble chromatin with a size range of 0.5 kb to 0.9 kb was prepared by sonication using a Bioruptor (Diagenode, Liège, Belgium) After centrifugation to remove cell debris, chromatin was pre-cleared (1 h at 4°C with a 50% gel slurry of protein A/G – agarose beads saturated with salmon sperm DNA and bovine serum albumin (Upstate, Billerica, MA), diluted in immunoprecipitation dilution buffer (0.01% SDS, 0.5% Triton X-100, 2 mM EDTA, 16.7 mM Tris-HCl pH 8.1, 100 mM NaCl and 1x protease inhibitor cocktail), and 10% of the supernatant was used as input. The diluted chromatin was incubated overnight at 4°C with the 2-5 µg of IgG, JMJD3 and H3K27me3, antibodies and the immune complexes were recovered by 1 h incubation at 4°C with a 50% gel slurry of protein A/G – agarose beads (Upstate). The precipitated complexes were washed sequentially with low salt buffer (0.1% SDS, 1% Triton X-100, 2 mM EDTA, 20 mM Tris-HCl pH 8.1, 150 mM NaCl and 1X protease inhibitor cocktail), high salt buffer (0.1% SDS, 1% Triton X-100, 2 mM EDTA, 20 mM Tris-HCl pH 8.1, 500 mM NaCl and 1X protease inhibitor cocktail), LiCl buffer (1 mM EDTA, 10 mM Tris-HCl pH 8.1, 250 mM LiCl, 1% Igepal, 1% deoxycholic acid and 1X protease inhibitor cocktail) and twice with Tris-EDTA buffer (1 mM EDTA and 20 mM Tris-HCl pH 8.1), and extracted twice with freshly prepared elution buffer (100 mM NaHCO_3_ and 1% SDS) with mild vortexing. The cross-linking between DNA and proteins was reversed by incubation with 0.3 M NaCl, overnight at 67°C, in the presence of RNase A. Samples were then digested with proteinase K at 45°C for 1 h. DNA was purified using QIAquick PCR purification kit (Qiagen Inc.) and analyzed by real-time PCR.

### Real-time PCR

Real-time PCRs were performed using SYBR green Master Mix in an ABI 7300 (Applied Biosystems, Carlsbad, CA) sequence detection system. The transcript expression of ARF and p53 were investigated using the following set of primers: ARF sense 5′- GCCGCACCGGAATCCT- 3′; ARF antisense 5′-TTGAGCAGAAGAACTGCTGCTACGT-3′; p53 sense 5′-GTGAAGCCCTCCGAGTGTCAGGAGC-3′; p53 antisense 5′-GGTGGGCAGCGCTCTCTTTGCGC-3′; GAPDH sense 5′- ATTCAACGGCACAGTCAAGG-3′; and GAPDH antisense 5′-TGGATGCAGGGATGATGTTC-3′. The product of the GAPDH RNA was used for endogenous normalization.

For ChIP experiments, real-time PCR was performed using primers that covered different regions of Arf gene, and the Hoxc8 promoter. The primers used were: 5′ GAC CGT GAA GCC GAC CCC TTC AGC 3′ (forward) and 5′ GGG GTC GCT TTC CCC TTC GG 3′ (reverse) for the Arf promoter, 5′ TGT GAC AAG CGA GGT GAG AAG C-3′ (forward) and 5′ ATG GGC GTG GAG CAA AGA TG 3′ (reverse) for the Arf exon1, and 5′ CCG GGA GTC TGA GGA ATT CGC 3′ (forward) and 5′ GGA CCG AAC CCC AAG CTG GC 3′ (reverse) for the Hoxc8 promoter. All PCR signals from immunoprecipitated DNA were normalized to PCR signals from non-immunoprecipitated input DNA. Results are first expressed as percentage of total input and converted to fold-change over IgG. Calculations take into account the values of at least six independent experiments.

### Densitometry and Statistical Analysis

The relative intensities of protein and nucleic acid bands were analyzed using the Quantity One Version 4.6 densitometric analysis program (Bio-Rad Laboratories). Results from different groups were compared using the Student's T-test, or one-way ANOVA. Kruskal-Wallis or the Mann-Whitney U tests were also used whenever the assumptions of the parametric test were not satisfied. Values of *p*<0.05 were considered statistically significant. All statistical analysis was performed with GraphPad InStat software (GraphPad Software, Inc, San Diego, CA).

## Results

### Modulation of p53 by JMJD3 during Differentiation of Mouse NSCs

**Figure 1:**
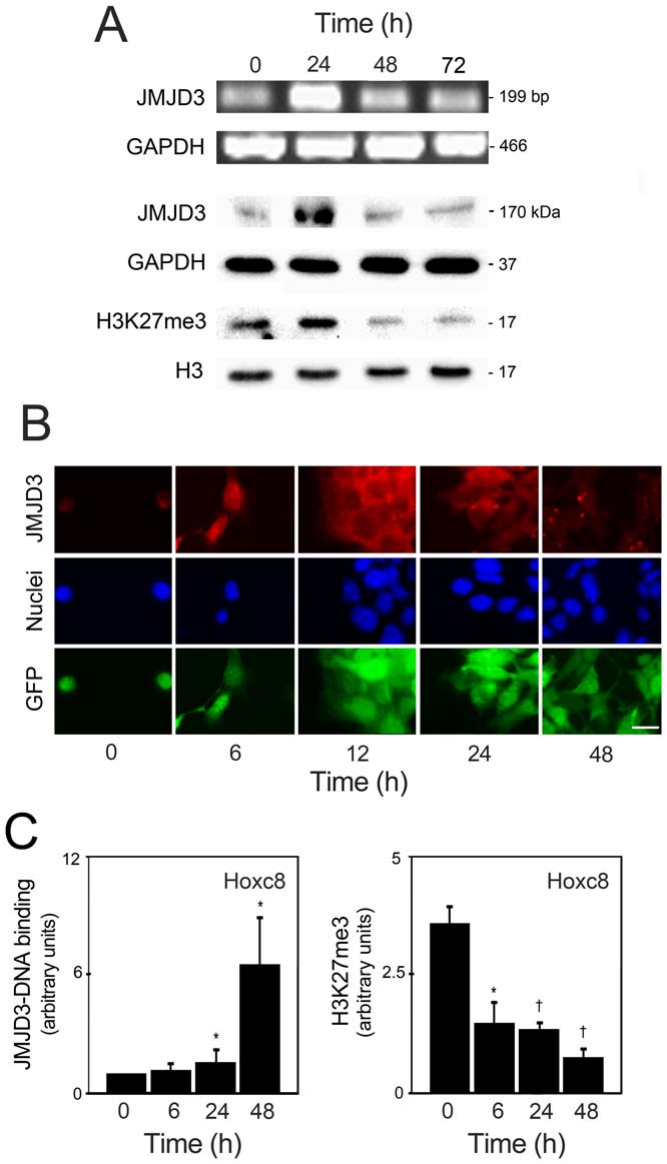
Endogenous JMJD3 expression is increased in early stage neural differentiation. Undifferentiated and differentiated mouse NSCs were collected for RT-PCR, Western blot, immunostaining and ChIP experiments at indicated times. *A*, JMJD3 mRNA expression (*top*), JMJD3 protein abundance (*middle*), and H3K27me3 histone modification (*bottom*) representative of at least 3 independent experiments. GAPDH and H3 were used as loading controls. *B*, Fluorescence staining of JMJD3 representative of at least 3 independent experiments. Scale, 10 µm. *C*, Presence of JMJD3 and the histone mark H3K27me3 at Hoxc8 promoter region in mouse NSCs under differentiation. The results were calculated by assigning the arbitrary value 1 to the DNA binding of IgG results, and expressed as mean ± SEM arbitrary units of 6 independent experiments. *p<0.05 and †*p*<0.01 from IgG controls.

JMJD3 controls the expression of key regulators and markers of neurogenesis, and is required for commitment to the neural lineage [13]. In addition, p53 has been described as a limiting factor of stem cell proliferative competence, playing a crucial role during neurogenesis [25]. In fact, p53 suppresses pluripotency and cellular dedifferentiation [26]. Although both JMJD3 and p53 have been implicated in neurogenesis of stem cells, the possible molecular interaction between the two has not yet been explored. We have previously characterized neural differentiation of mouse NSCs, where neurogenesis and gliogenesis occur at ∼3 and 8 days of differentiation, respectively [2], [27]. In the present study, we first evaluated the endogenous levels of JMJD3 throughout neural differentiation by RT-PCR, Western blot and immunocytochemistry. Our results showed that JMJD3 mRNA and protein levels significantly increased at 1 day of mouse NSC differentiation (*p*<0.05). In addition, JMJD3 activity was increased as detected by a significant decrease in trimethylation state of H3K27me3, visualized by Western blot (Fig. 1A).

Immunocytochemistry analysis confirmed RT-PCR and Western blot data, revealing that JMJD3 expression is already present in undifferentiated cells, and markedly increased at early stages of neural differentiation (Fig. 1B). Since it has been demonstrated that H3K27 demethylase activity of UTX and JMJD3 act at Hox gene promoters to derepress Hox gene transcription [28], [29], [30], we analyzed whether JMJD3 was present on the Hoxc8 promoter region during first stages of mouse NSC differentiation. ChIP experiments revealed that JMJD3 was present on Hoxc8 promoter region. Notably, immediately after differentiation, a significant loss of H3K27me3 was observed at Hoxc8 region (*p*<0.05) (Fig. 1C). These results corroborate JMJD3 activation throughout mouse NSC differentiation and confirm previous evidence showing that JMJD3 functions as a transcriptional activator by removing the H3K27me3/me2 marks [28], [29], [30].

**Figure 2:**
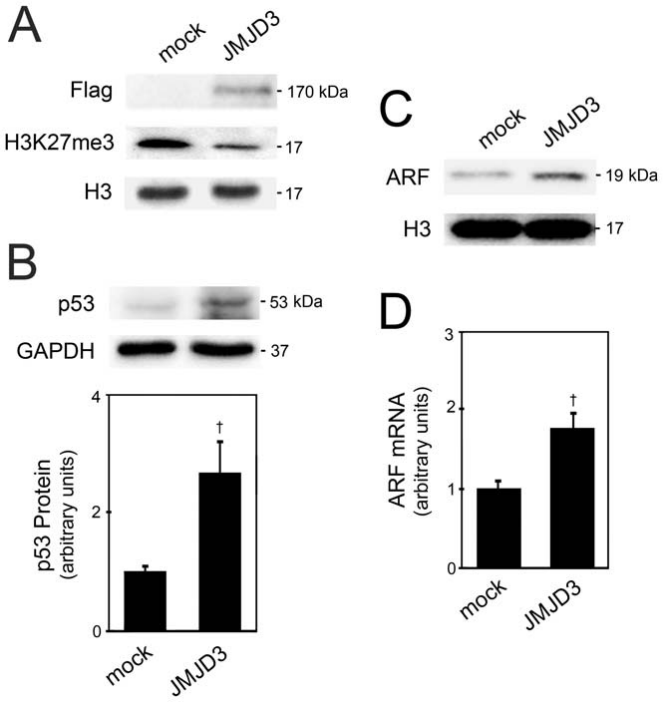
JMJD3 induces p53 stabilization during neural differentiation. Mouse NSCs were transfected with Flag-JMJD3 overexpression plasmid 3 h after induction of differentiation, and collected for Western blot analysis as described under “Material and Methods”. *A*, Representative immunoblots of Flag levels and H3K27me3, in control (mock) and JMJD3 overexpressing cells at 24 h of differentiation. H3 was used as loading control. *B*, Representative immunoblots of p53 total levels in control (mock) and JMJD3 overexpressing cells (*top*) and corresponding histograms (*bottom*) at 48 h of differentiation. GAPDH was used as loading control. *C*, Representative immunoblot of ARF total levels in control (mock) and JMJD3 overexpressing cells at 48 h of differentiation. H3 was used as loading control. *D*, Histogram of ARF mRNA total levels in JMJD3 overexpressing cells. The results are expressed as mean ± SEM arbitrary units of at least 4 independent experiments. †*p*<0.01 and ‡*p*<0.001 from controls.

To clarify whether JMJD3 regulates p53 expression during neurogenesis, we overexpressed JMJD3 by transfecting mouse NSCs with a Flag-JMJD3 plasmid and evaluated both mRNA and protein levels of p53 after 48 h of differentiation. To determine the cellular effects of JMJD3 overexpression, H3K27me3 was also measured by Western blot 24 h after transfection (Fig. 2A). Flag-JMJD3 overexpression decreased H3K27 trimethylation by ∼50%, when compared with control (mock) cells (*p*<0.05). More importantly, overexpression of JMJD3 significantly increased p53 protein levels during differentiation of mouse NSCs (*p*<0.01) (Fig. 2B). Nevertheless, real-time RT-PCR showed that p53 mRNA was significantly reduced in these conditions (*p*<0.001), indicating that JMJD3 does not directly increase p53 transcription (data not shown). JMJD3-induced downregulation of p53 mRNA might result from negative feedback mechanisms of p53 autoregulation [31]. Therefore, these data suggest that JMJD3 induces p53 stabilization during differentiation of NSCs. A potential interaction between p53 and JMJD3 has been demonstrated in mouse embryonic fibroblasts, where JMJD3 regulates p53 levels through ARF [15], [16]. This mechanism was reported during senescence of differentiated cells, but has not been described during neural differentiation. To clarify the role of JMJD3 in p53 stabilization and the potential involvement of ARF, we measured ARF mRNA and protein levels during mouse NSC differentiation. Western blot and real-time RT-PCR analysis showed that JMJD3 overexpression resulted in ∼2-fold increased ARF mRNA and protein levels (*p*<0.01) (Fig. 2C and D), coinciding with p53 protein accumulation (Fig. 2B).

### Presence of JMJD3 Binding at the ARF Locus during Mouse NSC Differentiation

**Figure 3:**
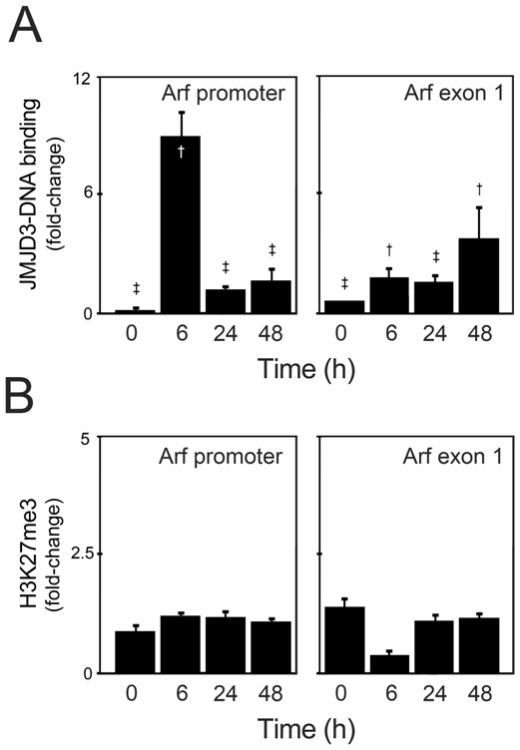
Enhanced recruitment of JMJD3 to the ARF locus during neural differentiation. Mouse NSCs were subjected to ChIP analysis at 0, 6, 24 and 48 h after differentiation, using antibodies and primer sets as described under “Material and Methods”. *A*, Presence of JMJD3 at both promoter and exon 1 regions of ARF locus in mouse NSCs under differentiation. Relative levels of JMJD3 immune complex were calculated by assigning the arbitrary value 1 to IgG levels. *B*, Absence of the histone mark H3K27me3 at promoter and exon 1 regions of ARF locus in mouse NSCs under differentiation. Hoxc8 was used as positive control. The results are expressed as mean ± SEM fold change of 6 independent experiments. *p<0.05, †*p*<0.01 and ‡*p*<0.001 from IgG controls.

To further investigate the role of ARF in JMJD3-induced p53 protein stabilization, we used ChIP assays and evaluate the occupancy of JMJD3 on the ARF promoter during differentiation of mouse NSCs (Fig. 3). Following immunoprecipitation with JMJD3 or control (IgG) antibodies, real-time PCR was performed using primers for different regions of ARF, such as ARF promoter and ARF exon 1, as well as primers for the Hoxc8 promoter region, as a positive control. Interestingly, our results showed a time-dependent increase in the occupancy of JMJD3 at the ARF locus during neural differentiation (Fig. 3A). In fact, association of JMJD3 with the ARF promoter was strong at 6 h of neural differentiation (*p*<0.01), and still observed at 24 and 48 h of differentiation (*p*<0.001). Nevertheless, after 6 h of neural induction, JMJD3 occupancy was greater at ARF exon 1, as compared with ARF promoter.

To clarify whether the presence of JMJD3 was associated with demethylation of H3K27me3 in ARF locus during neural differentiation, we analyzed the levels of trimethylated state H3K27 in both ARF promoter and exon 1 regions throughout mouse NSC differentiation (Fig. 3B). Although increased levels of H3K27me3 were detected at Hoxc8 promoter region in undifferentiated NSCs (Fig. 1C), H3K27me3 was not detected on either ARF promoter or exon 1 regions, and levels of H3K27me3 were unchanged throughout differentiation. These data directly implicate JMJD3 in the induction of ARF expression during neural differentiation, although independently of histone demethylation.

### JMJD3 Directly Interacts with p53 during Mouse NSC Differentiation

**Figure 4:**
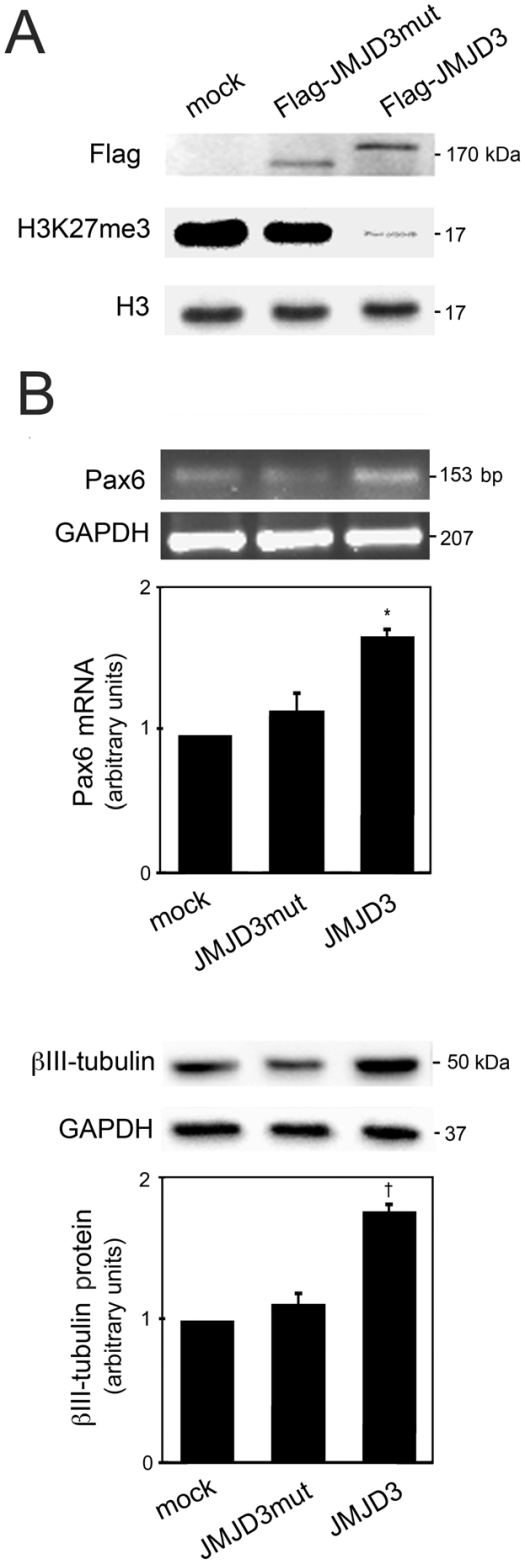
JMJD3-induced demethylation accelerates neurogenesis. Mouse NSCs were transfected with either Flag-JMJD3 or Flag-JMJD3mut overexpression plasmids 3 h after induction of differentiation, and total proteins were extracted for Western blot and RT-PCR assays, as described under “Material and Methods”. *A*, Representative immunoblots with Flag- and H3K27me3-specific antibodies in control (mock), JMJD3mut and JMJD3 overexpressed cells. *B*, Representative RT-PCR and immunoblot and corresponding histograms of total levels of Pax6 (*top*) and β-III tubulin (*bottom*) during mouse NSC differentiation. All densitometry values were normalized to GAPDH expression, and the results expressed as mean ± SEM arbitrary units of at least 3 independent experiments. *p<0.05 and †*p*<0.01 from controls.

Based on previous results and the available literature on the effects of JMJD3 in a H3K27 demethylation-independent manner [32], we evaluated specific neural markers in mouse NSCs overexpressing Flag-JMJD3 or Flag-JMJD3mut, which does not contain the C-terminal region associated with demethylase activity (Fig. 4). While levels of H3K27me3 in control (mock) cells and in cells transfected with Flag-JMJD3mut overexpression plasmid were similar, H3K27me3 levels in cells transfected with Flag-JMJD3 were dramatically reduced, consistent with a role for JMJD3 as H3K27 demethylase (Fig. 4A). Interestingly, our results revealed that JMJD3 overexpression significantly increased several neural markers, including the neural precursor Pax6 (*p*<0.05) and the neuronal progenitor β-III tubulin (*p*<0.01) (Fig. 4B). In fact, 2 days after induction of neural differentiation, Pax6 mRNA expression was higher in cells transfected with wild-type JMJD3, when compared with mock and JMJD3mut-transfected cells. The expression of β-III tubulin was also higher in cells transfected with wild-type JMJD3. These results are consistent with a role of JMJD3-dependent demethylation in neurogenesis progression.

**Figure 5:**
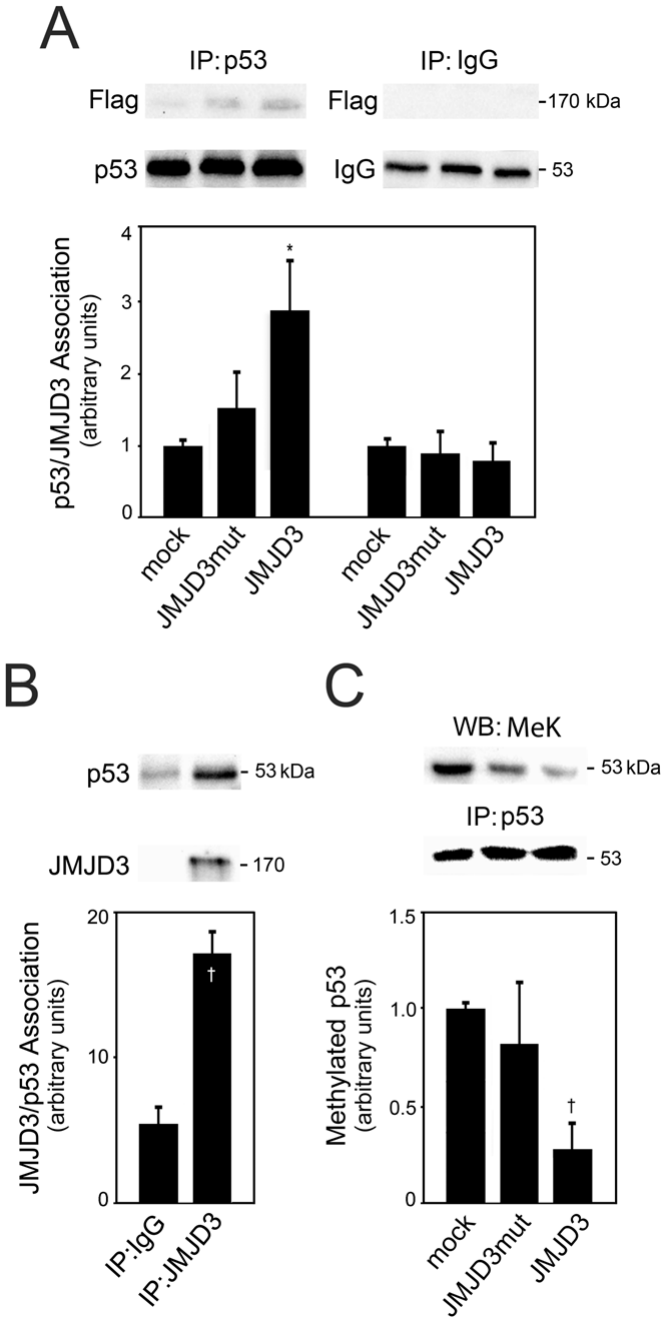
JMJD3 directly associates with p53 during neural differentiation. Mouse NSCs were transfected with either Flag-JMJD3 or Flag-JMJD3mut overexpression plasmids 3 h after induction of differentiation. Total proteins were collected for immunoprecipitation assays as described under “Material and Methods”. *A*, Representative immunoblots with Flag- and p53-specific antibodies (*top*) and histogram of p53/Flag-JMJD3 association (*bottom*). *B*, Representative immunoblots with JMJD3- and p53-specific antibodies (*top*) and histogram of endogenous JMJD3/p53 association (*bottom*). *C*, Representative immunoblots with methylated lysine- and p53-specific antibodies (*top*), and histogram of methylated levels of p53 (*bottom*). All densitometry values for Flag and methylated lysines were normalized to the respective p53 expression, and the results expressed as mean ± SEM arbitrary units of at least 3 independent experiments. *p<0.05 and †*p*<0.01 from controls. IP, Immunoprecipitation; WB, Western blot; MeK, Methyl K pan.

Since p53 is a non-histone target for several histone demethylases [21], [22], we hypothesized that JMJD3 may directly interact with p53 in addition to regulating ARF and thus p53 protein levels, influencing the pro-neurogenic function of p53. To investigate the potential JMJD3/p53 physical association, we performed immunoprecipitation assays in mouse NSCs overexpressing Flag-JMJD3 or Flag-JMJD3mut, at 2 days of neural differentiation. After immunoprecipitation of p53 or IgG, Western blot analysis revealed a significant increase in p53/JMJD3 association in cells transfected with Flag-JMJD3 overexpression plasmids (*p*<0.05), suggesting a direct interaction between these two proteins (Fig. 5A). There was also a slight, but not significant, increase in the association between p53 and JMJD3 in cells transfected with Flag-JMJD3mut relative to control (mock), suggesting that the interaction between p53 and JMJD3 does not depend on the catalytic JmjC domain of JMJD3. Importantly, immunoprecipitation assays demonstrated an association between p53 and endogenous JMJD3, in non-transfected cells, relative to control (IgG) (*p*<0.001) (Fig. 5B).

To better characterize the molecular interaction between JMJD3 and p53, lysates from cells overexpressing Flag-JMJD3 or Flag-JMJD3mut were immunoprecipitated with anti-p53 antibodies and subjected to Western blotting with anti-methylated lysine antibodies (Fig. 5C). p53 was significantly less methylated in cells expressing wild-type JMJD3, as compared to cells transfected with the C-terminal mutant form of JMJD3 or control (mock) cells (*p*<0.01). This suggests that the proneural effects of p53 may be associated with a direct JMJD3-dependent demethylation.

### JMJD3 Induces p53 Nuclear Distribution in a Demethylase-dependent Manner

**Figure 6:**
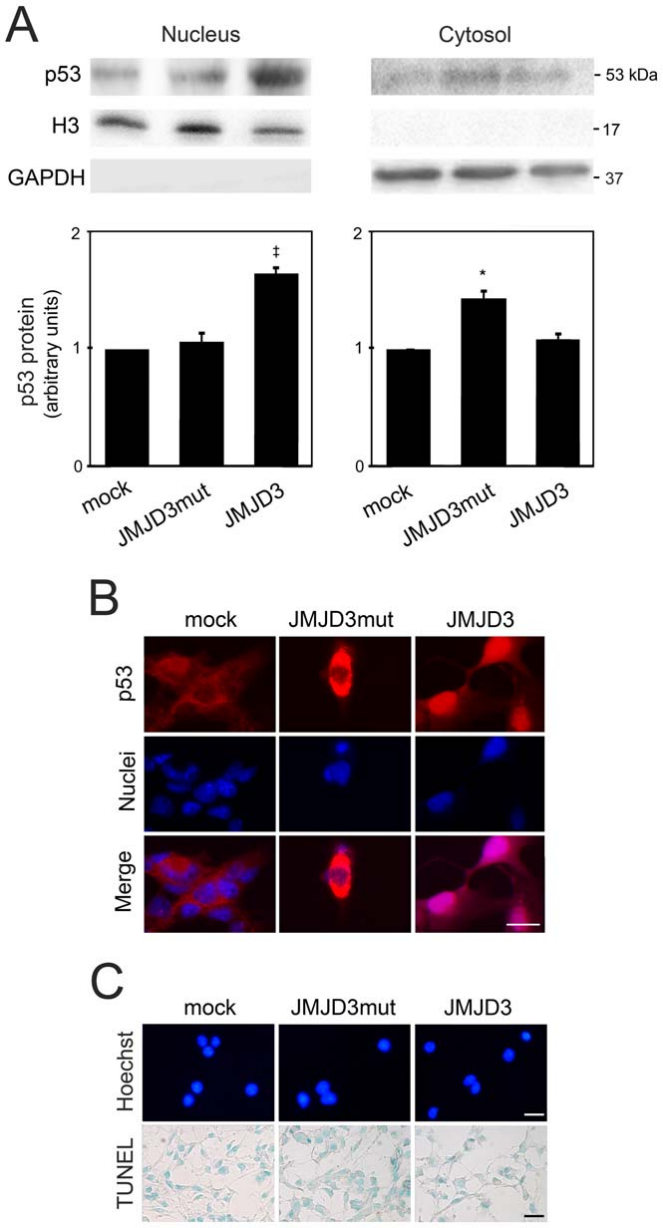
JMJD3 induces p53 nuclear trafficking in a demethylase-dependent manner. Mouse NSCs were transfected with either JMJD3 or JMJD3mut overexpression plasmids 3 h after induction of differentiation, and collected for Western blot, immunocytochemistry and apoptosis assays, as described under “Material and Methods”. *A*, Representative immunoblots of nuclear and cytosolic levels of p53 (*top*) and corresponding histograms (*bottom*) in control (mock), JMJD3mut and JMJD3 overexpressing cells. GAPDH and H3 were used as loading controls. Results are expressed as mean ± SEM arbitrary units of at least 4 independent experiments. *p<0.05 and ‡*p*<0.001 from controls. *B*, Subcellular localization of p53 in mouse NSCs transfected with either JMJD3 or JMJD3mut overexpression plasmids. *C*, Evaluation of apoptosis by Hoechst staining and TUNEL assay. Representative images from at least 3 independent experiments. Scale, 10 µm.

p53 controls cell fate through trafficking among organelles, which is modulated by PTMs [33]. The catalytic JmjC domain may directly demethylate p53 and control its trafficking and subcellular distribution, thus influencing its retention or translocation to the nucleus. *In vitro* experiments in models of neural maturation, and *in vivo* analysis of axonal injury and regeneration suggest that some “atypical” p53-dependent cellular functions could depend on specific patterns of p53 post-translation modifications. These modifications directly affect p53 subcellular localization, transcriptional activity, and affinity to diverse cofactors [34]. Therefore, we investigated the effect of JMJD3 demethylase activity on p53 subcellular localization (Fig. 6). Overexpression of wild-type JMJD3 resulted in ∼65% increased nuclear p53 during NSC differentiation (*p*<0.001), while overexpression of Flag-JMJD3mut, which lacks demethylase activity, caused cytoplasmatic accumulation of p53 (>40%; *p*<0.05) (Fig. 6A). To confirm these results, we performed immunocytochemistry assays on differentiated mouse NSCs transfected with wild-type and mutant JMJD3 (Fig. 6B). Indeed, p53 immunofluorescence also revealed an accumulation of nuclear p53, in the presence of wild-type JMJD3, while p53 in cells overexpressing Flag-JMJD3mut was largely cytosolic. Thus, JMJD3 modulates cellular distribution of p53 in a demethylase activity-dependent manner. Importantly, these results were not associated with an increase in cell death, as visualized by Hoechst staining and confirmed by the TUNEL assay (Fig. 6C).

## Discussion

H3K27 demethylase JMJD3 controls the expression of key regulators and markers of neurogenesis, and is required for commitment to the neural lineage [13], [35], [36]. Nevertheless, the precise mechanisms by which JMJD3 triggers signaling pathways involved in the control of neurogenesis are not fully understood. In addition, JMJD3 has recently been shown to act as a tumor suppressor by activating ARF and eliciting p53-dependent arrest in oncogene-induced senescence using mouse and human fibroblasts [15]. The present study identifies a new molecular link between proapoptotic p53 and JMJD3 demethylase, in the context of neural differentiation. JMJD3-induced p53 stabilization appears to be mediated by ARF and, more importantly, p53 demethylation by JMJD3 results in its nuclear accumulation.

p53 is a tumor suppressor protein that induces cell cycle arrest or apoptosis in response to cellular stresses [37]. p53 plays a crucial role in eliciting neuronal cell death during development, and in adult organisms after exposure to a range of stressors and/or DNA damage. However, non-apoptotic roles of p53 have emerged during the past few years, describing p53 as an important player of cell fate decisions. Following cell stress, p53 may undergo numerous PTMs that result in different cellular outcomes, such as neural survival and regeneration. Indeed, p53 PTMs might promote neuronal maturation, as well as axon outgrowth and regeneration, after neuronal injury [11]. This leading role of p53 contributes to directing neurons toward a specific phenotype in critical conditions, such as during development and following cellular damage [38].

Recently, p53 has been shown to be key in suppressing pluripotency and cellular dedifferentiation [26]. p53 and Pten inactivation cooperate to increase Myc expression, thus inducing high-grade malignant gliomas. Attenuated Myc expression, in turn, appears to restore neural differentiation and reduce tumorigenic potential [3]. Others have shown that p53 is a limiting factor of stem cell proliferative competence, playing a crucial role during neurogenesis [25], [39]. Nevertheless, the proneural effect of p53 is still a controversial issue. In NSCs obtained from the olfactory bulb of embryonic mice, it has been shown that lack of p53 favors neural differentiation. However, a positive correlation between cell density and neuron percentage was found, and an enrichment of neurosphere-forming cells in p53KO mice embryos was described [39]. Moreover, it has been suggested that although loss of p53 by itself is not sufficient for tumor formation, it can provide a proliferative advantage to the slow- and fast-proliferating subventricular zone stem cell populations associated with their rapid differentiation [40]. In this regard, loss of p53 is also associated with an increased number of adult NSCs and neuroblasts. Thus, the precise role of p53 during neurogenesis, namely whether neurogenesis induced by the absence of p53 is only the ultimate result of increased number of neural progenitors remains to be further investigated. Finally, other studies have reported that activation of p53 blocks epigenetic reprogramming [41]. In fact, ablation of different senescence effectors, such as p53, p16^INK4a^ and p21^CIP1^ improves the efficiency of reprogramming somatic cells into induced-pluripotent stem (iPS) cells [42]. Reducing p53 expression markedly enhances reprogramming efficiency and prevents chromatin condensation. These evidences corroborate the crucial role of p53 in regulating differentiation and development.

We have recently explored the role of p53 during neural differentiation, and demonstrated that p53 interferes with the Akt/p-FOXO3A/Id1 survival pathway in mouse NSCs , while silencing of p53 leads to a delay in neurogenesis, but not in gliogenesis [2]. Although we did not find a significant modulation of total p53 total protein levels from 1 to 8 days of differentiation in our previous study, p53 may still need to be stabilized during this process. In fact, p53 activity is increased at 3 days of differentiation and this is not associated with increased cell death, adding differentiation to the spectrum of p53-based cell fate decisions. Based on the role of p53 and JMJD3 during neural differentiation as well as on the above evidence, we investigated a possible molecular mechanism of interaction between p53 and JMJD3 in the context of neurogenesis.

In this study, we first demonstrated that expression of active JMJD3 is increased and activated at early stages of differentiation in mouse NSCs, inducing ARF transcriptional activation and p53 protein stabilization. ChIP analysis revealed that JMJD3 occupied the ARF promoter region at 6 h following neural differentiation, which was consistent with immunocytochemistry analysis showing induction of JMJD3 as early as 6 h. Indeed, a role for ARF in PcG-mediated stem cell cycle control has been suggested by others. In a neurosphere population, Bmi1-mediated ARF/p53 repression plays a general role in curtailing proliferation, implicating PcG proteins not only in embryonic developmental fate decisions, but also in discriminative processes between cell cycle control of stem- and more differentiated cells [19]. In this example, repression of ARF is required for neurosphere self-renewal. Others have proposed that methylation in the promoter region of the ARF gene may be used as a biomarker for the diagnosis of gliomas [43]. Interestingly, in contrast with what happens in mouse embryonic fibroblasts, we found that JMJD3 does not primarily induce demethylation of the ARF gene during neurogenesis. Nevertheless, it is possible that JMJD3 erases H3K27me3, the histone mark associated with transcriptional repression, on the ARF gene at later time-points, not totally excluding the role of JMJD3 demethylase function in ARF transcriptional activation in this cellular context. In fact, others have shown that the recruitment of JMJD3 during neural development induces an increase in Pax6 transcription, a homeodomain transcription factor that controls the differentiation of the radial glia, before exerting its function of H3K27 demethylase on its promoter [13]. This suggests that JMJD3 first contributes to Pax6 activation through mechanisms that are independent of its H3K27me3 demethylase activity, or that are mediated by indirect mechanisms. Moreover, JMJD3 fine-tunes the transcriptional output of lipopolysaccharide-activated macrophages in an H3K27 demethylation-independent manner [32]. Therefore, it is possible that JMJD3 modulates ARF expression in the context of neural differentiation primarily as a transcription factor. We have also found that the C-terminal mutant form of JMJD3 induced ARF expression in mouse NSCs (data not shown), supporting the idea that demethylase activity of JMJD3 is not a prerequisite for ARF transcriptional activation in neural differentiation. Our data also revealed that overexpression of JMJD3 accelerates neurogenesis, enhancing efficient expression of neuron-specific markers, including βIII-tubulin. These data validate the role of JMJD3 in modulating neural differentiation through a demethylase activity-dependent mechanism. In fact, it has been shown that the inactivation of PcG by knockout of Ring1B or Ezh2 gene, or knockdown of Eed prolonged the neurogenic phase of neural precursor cells and delayed the onset of the astrogenic phase. Moreover, PcG repress the promoter of the proneural gene neurogenin1 in a developmental-stage-dependent manner. These results demonstrated the role of PcG as temporal regulator of neural fate [44]. To further investigate a possible molecular link between JMJD3 and p53 in the context of neurogenesis, we evaluated their psychical interaction and the effect of JMJD3 demethylase function on p53 methylation levels in mouse NSCs undergoing differentiation. Our data showed that p53 and JMJD3 directly interact after neural differentiation, and the presence or absence of JMJD3 demethylase function appears to differentially modulate the levels of p53 lysine methylation. Curiously, JmjC proteins can also demethylate arginine residues and, at least in theory, other protein substrates or nucleotides. In fact, JmjC proteins are also found in bacteria that do not contain histones, suggesting that these proteins have other functions besides histone demethylation [45].

p53 plays a heterogeneity of functions promoted by the diversity of molecular targets of p53 itself, which in turn are determined by different PTMs that p53 may suffer [21], [46]. Accordingly, the complex mechanism through which p53 decides between cell death or differentiation molecular targets could be justified by PTMs. In the present study, we evaluated whether the JMJD3-induced p53 demethylation regulates the cellular localization of p53 during differentiation of mouse NSCs. Notably, our results showed a regulation of p53 nuclear and cytosolic levels by JMJD3 through a demethylase-dependent mechanism. Our findings, coupled with others showing that regulation of p53 through lysine methylation affects p53 cellular function and distribution [47] are interesting, as they identify a new lysine demethylase protein as a direct regulator of p53 function during neural differentiation. Here we show that although the apoptotic functions of p53 are also associated with p53 nuclear localization, JMJD3-induced p53 demethylation and nuclear translocation during neural differentiation did not result in higher levels of cell death. However, it remains to be determined which specific residue(s) within p53 is modified by JMJD3 in this particular cellular context. Finally, it would be interesting to investigate the specific direct targets of JMJD3-dependent demethylation of p53 during neurogenesis, to clarify whether they include ARF itself.

Collectively, our results provide an extended mechanism of action for JMJD3, in which this demethylase binds to and regulates p53 methylation status, inducing significant accumulation of p53 in the nucleus of NSCs undergoing differentiation. This is not associated with apoptotic cell death, but may influence the neurogenic process in mouse NSCs. Finally, a further insight into JMJD3-dependent p53 modulation of these different mechanisms is necessary to elucidate the decision-making processes between neuronal cell death and differentiation.

## Supporting Information

Figure S1 **p53 expression in p53-silenced (siRNA) mouse NSCs.** Cells were incubated with either control or p53 siRNA and collected after 2 days in differentiation medium. Total proteins were extracted for immunoblot analysis. Representative immunoblots of p53 and β-actin in cells transfected with either control or p53 siRNA.
(TIF)

## References

[1] Fujita J, Crane AM, Souza MK, Dejosez M, Kyba M (2008). Caspase activity mediates the differentiation of embryonic stem cells.. Cell Stem Cell.

[2] Aranha MM, Sola S, Low WC, Steer CJ, Rodrigues CM (2009). Caspases and p53 modulate FOXO3A/Id1 signaling during mouse neural stem cell differentiation.. J Cell Biochem.

[3] Zheng H, Ying H, Yan H, Kimmelman AC, Hiller DJ (2008). p53 and Pten control neural and glioma stem/progenitor cell renewal and differentiation.. Nature.

[4] Di Giovanni S, Knights CD, Rao M, Yakovlev A, Beers J (2006). The tumor suppressor protein p53 is required for neurite outgrowth and axon regeneration.. Embo J.

[5] Hong H, Takahashi K, Ichisaka T, Aoi T, Kanagawa O (2009). Suppression of induced pluripotent stem cell generation by the p53-p21 pathway.. Nature.

[6] Li H, Collado M, Villasante A, Strati K, Ortega S (2009). The Ink4/Arf locus is a barrier for iPS cell reprogramming.. Nature.

[7] Kawamura T, Suzuki J, Wang YV, Menendez S, Morera LB (2009). Linking the p53 tumour suppressor pathway to somatic cell reprogramming.. Nature.

[8] Utikal J, Polo JM, Stadtfeld M, Maherali N, Kulalert W (2009). Immortalization eliminates a roadblock during cellular reprogramming into iPS cells.. Nature.

[9] Marion RM, Strati K, Li H, Murga M, Blanco R (2009). A p53-mediated DNA damage response limits reprogramming to ensure iPS cell genomic integrity.. Nature.

[10] Riley T, Sontag E, Chen P, Levine A (2008). Transcriptional control of human p53-regulated genes.. Nat Rev Mol Cell Biol.

[11] Tedeschi A, Di Giovanni S (2009). The non-apoptotic role of p53 in neuronal biology: enlightening the dark side of the moon.. EMBO Rep.

[12] Meissner A, Mikkelsen TS, Gu H, Wernig M, Hanna J (2008). Genome-scale DNA methylation maps of pluripotent and differentiated cells.. Nature.

[13] Burgold T, Spreafico F, De Santa F, Totaro MG, Prosperini E (2008). The histone H3 lysine 27-specific demethylase Jmjd3 is required for neural commitment.. PLoS One.

[14] Sawarkar R, Paro R (2010). Interpretation of developmental signaling at chromatin: the polycomb perspective.. Dev Cell.

[15] Barradas M, Anderton E, Acosta JC, Li S, Banito A (2009). Histone demethylase JMJD3 contributes to epigenetic control of INK4a/ARF by oncogenic RAS.. Genes Dev.

[16] Agger K, Cloos PA, Rudkjaer L, Williams K, Andersen G (2009). The H3K27me3 demethylase JMJD3 contributes to the activation of the INK4A-ARF locus in response to oncogene- and stress-induced senescence.. Genes Dev.

[17] Harris SL, Levine AJ (2005). The p53 pathway: positive and negative feedback loops.. Oncogene.

[18] Nagao M, Campbell K, Burns K, Kuan CY, Trumpp A (2008). Coordinated control of self-renewal and differentiation of neural stem cells by Myc and the p19ARF-p53 pathway.. J Cell Biol.

[19] Bruggeman SW, Valk-Lingbeek ME, van der Stoop PP, Jacobs JJ, Kieboom K (2005). Ink4a and Arf differentially affect cell proliferation and neural stem cell self-renewal in Bmi1-deficient mice.. Genes Dev.

[20] Allison SJ, Milner J (2003). Loss of p53 has site-specific effects on histone H3 modification, including serine 10 phosphorylation important for maintenance of ploidy.. Cancer Res.

[21] Scoumanne A, Chen X (2008). Protein methylation: a new mechanism of p53 tumor suppressor regulation.. Histol Histopathol.

[22] Tsai WW, Nguyen TT, Shi Y, Barton MC (2008). p53-targeted LSD1 functions in repression of chromatin structure and transcription in vivo.. Mol Cell Biol.

[23] Reynolds BA, Weiss S (1992). Generation of neurons and astrocytes from isolated cells of the adult mammalian central nervous system.. Science.

[24] Rietze RL, Reynolds BA (2006). Neural stem cell isolation and characterization.. Methods Enzymol.

[25] Krizhanovsky V, Lowe SW (2009). Stem cells: The promises and perils of p53.. Nature.

[26] Zhao T, Xu Y (2010). p53 and stem cells: new developments and new concerns.. Trends Cell Biol.

[27] Aranha MM, Santos DM, Xavier JM, Low WC, Steer CJ (2010). Apoptosis-associated microRNAs are modulated in mouse, rat and human neural differentiation.. BMC Genomics.

[28] Agger K, Cloos PA, Christensen J, Pasini D, Rose S (2007). UTX and JMJD3 are histone H3K27 demethylases involved in HOX gene regulation and development.. Nature.

[29] Lan F, Bayliss PE, Rinn JL, Whetstine JR, Wang JK (2007). A histone H3 lysine 27 demethylase regulates animal posterior development.. Nature.

[30] Lee MG, Villa R, Trojer P, Norman J, Yan KP (2007). Demethylation of H3K27 regulates polycomb recruitment and H2A ubiquitination.. Science.

[31] Bond GL, Hu W, Levine AJ (2005). MDM2 is a central node in the p53 pathway: 12 years and counting.. Curr Cancer Drug Targets.

[32] De Santa F, Narang V, Yap ZH, Tusi BK, Burgold T (2009). Jmjd3 contributes to the control of gene expression in LPS-activated macrophages.. Embo J.

[33] Ferecatu I, Rincheval V, Mignotte B, Vayssiere JL (2009). Tickets for p53 journey among organelles.. Front Biosci.

[34] Sims RJ, 3rd, Reinberg D (2008). Is there a code embedded in proteins that is based on post-translational modifications?. Nat Rev Mol Cell Biol.

[35] Jones RS (2007). Epigenetics: reversing the ‘irreversible’.. Nature.

[36] Jepsen K, Solum D, Zhou T, McEvilly RJ, Kim HJ (2007). SMRT-mediated repression of an H 27 demethylase in progression from neural stem cell to neuron.. Nature.

[37] Amaral JD, Xavier JM, Steer CJ, Rodrigues CM (2010). Targeting the p53 pathway of apoptosis.. Curr Pharm Des.

[38] Helton ES, Chen X (2007). p53 modulation of the DNA damage response.. J Cell Biochem.

[39] Armesilla-Diaz A, Bragado P, Del Valle I, Cuevas E, Lazaro I (2009). p53 regulates the self-renewal and differentiation of neural precursors.. Neuroscience.

[40] Gil-Perotin S, Marin-Husstege M, Li J, Soriano-Navarro M, Zindy F (2006). Loss of p53 induces changes in the behavior of subventricular zone cells: implication for the genesis of glial tumors.. J Neurosci.

[41] Selvaraj V, Plane JM, Williams AJ, Deng W (2010). Switching cell fate: the remarkable rise of induced pluripotent stem cells and lineage reprogramming technologies.. Trends Biotechnol.

[42] Banito A, Rashid ST, Acosta JC, Li S, Pereira CF (2009). Senescence impairs successful reprogramming to pluripotent stem cells.. Genes Dev.

[43] He J, Qiao JB, Zhu H (2010). p14(ARF) promoter region methylation as a marker for gliomas diagnosis.. Med Oncol.

[44] Hirabayashi Y, Suzki N, Tsuboi M, Endo TA, Toyoda T (2009). Polycomb limits the neurogenic competence of neural precursor cells to promote astrogenic fate transition.. Neuron.

[45] Cloos PA, Christensen J, Agger K, Helin K (2008). Erasing the methyl mark: histone demethylases at the center of cellular differentiation and disease.. Genes Dev.

[46] Stiewe T (2007). The p53 family in differentiation and tumorigenesis.. Nat Rev Cancer.

[47] Chuikov S, Kurash JK, Wilson JR, Xiao B, Justin N (2004). Regulation of p53 activity through lysine methylation.. Nature.

