# Supplementary figures and images for: p53 Interaction with JMJD3 Results in Its Nuclear Distribution during Mouse Neural Stem Cell Differentiation
Source: PLoS One. 2011 Mar 31;6(3):e18421. doi: 10.1371/journal.pone.0018421 (PMC3069089; doi:10.1371/journal.pone.0018421)

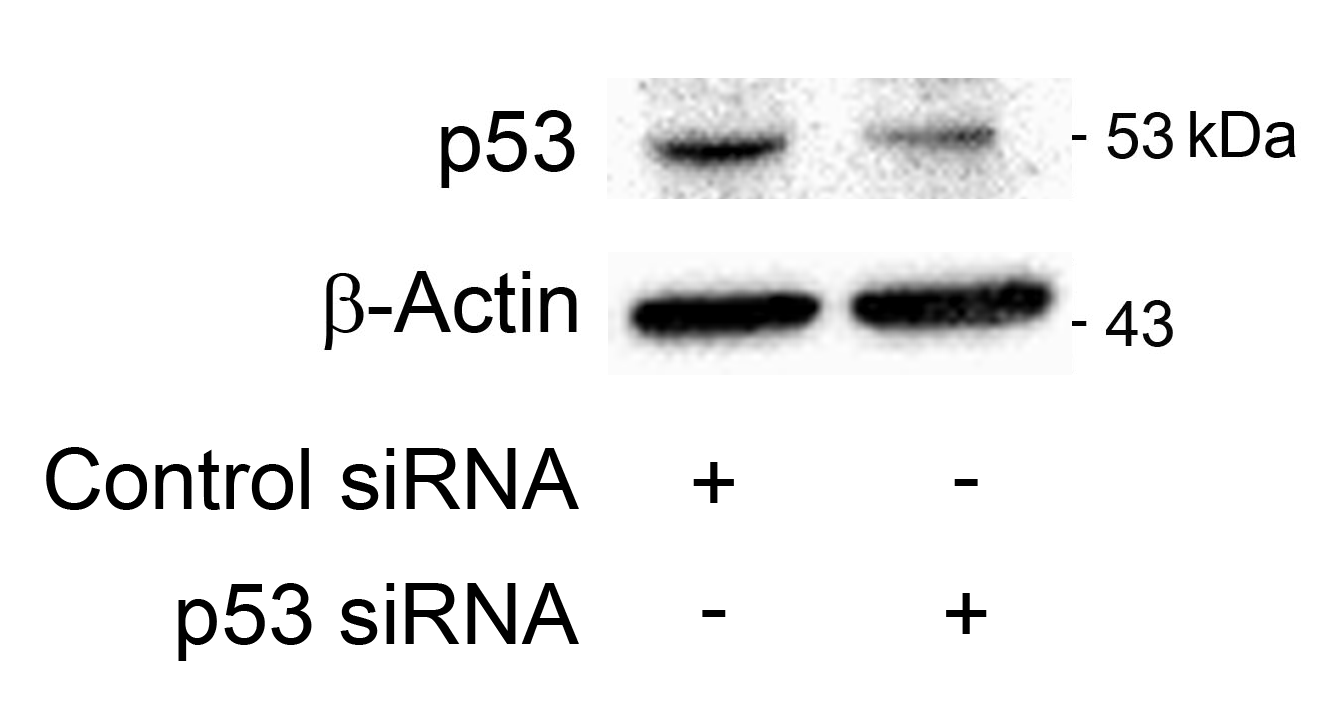

Supplement: Figure S1 — p53 expression in p53-silenced (siRNA) mouse NSCs. Cells were incubated with either control or p53 siRNA and collected after 2 days in differentiation medium. Total proteins were extracted for immunoblot analysis. Representative immunoblots of p53 and β-actin in cells transfected with either control or p53 siRNA. (TIF) [file pone.0018421.s001.tif]
